# Supplementary material for: CASPredict: a web service for identifying Cas proteins
Source: PeerJ. 2021 Jul 30;9:e11887. doi: 10.7717/peerj.11887 (PMC8327967; doi:10.7717/peerj.11887)
Supplement: Supplemental Information 4 [file peerj-09-11887-s004.docx]

167 optimal-features: ['YL', 'EK', 'RE', 'AS', 'LR', 'LK', 'RL', 'IY', 'DQ', 'VY', 'KY', 'YV', 'AA', 'YA', 'VA', 'YD', 'YK', 'AG', 'TM', 'EN', 'VM', 'MD', 'KD', 'GV', 'NR', 'VT', 'TA', 'WR', 'TS', 'TV', 'CV', 'KE', 'DG', 'QG', 'GQ', 'GH', 'AT', 'PQ', 'MT', 'SS', 'DK', 'VG', 'RI', 'HS', 'QP', 'ND', 'QT', 'WV', 'TG', 'MS', 'EY', 'IG', 'TT', 'TD', 'RR', 'TQ', 'LE', 'IK', 'KN', 'FK', 'KL', 'FE', 'PH', 'YY', 'CT', 'GI', 'QY', 'AP', 'GM', 'YF', 'ED', 'SC', 'LY', 'GS', 'FY', 'AI', 'SH', 'MH', 'KF', 'ST', 'AY', 'GG', 'SA', 'IM', 'KI', 'DD', 'MP', 'NY', 'GE', 'SQ', 'YS', 'PN', 'KK', 'GT', 'KR', 'SL', 'TP', 'NT', 'KS', 'SM', 'GC', 'EV', 'EL', 'WC', 'MA', 'HP', 'VR', 'CC', 'MM', 'LH', 'DY', 'SG', 'LM', 'CW', 'PM', 'GW', 'YN', 'GD', 'PS', 'YI', 'VQ', 'FT', 'LI', 'PC', 'EQ', 'QQ', 'RY', 'EE', 'EP', 'LS', 'AV', 'GP', 'IR', 'EI', 'FL', 'QS', 'AQ', 'YR', 'PD', 'KV', 'HA', 'AD', 'GK', 'PF', 'LD', 'SP', 'WQ', 'LT', 'TW', 'SY', 'LW', 'LG', 'HD', 'NK', 'GA', 'CH', 'HC', 'NV', 'IT', 'DA', 'DW', 'TN', 'TI', 'MR', 'KH', 'QL', 'VH']
