# Supplementary material for: CASPredict: a web service for identifying Cas proteins
Source: PeerJ. 2021 Jul 30;9:e11887. doi: 10.7717/peerj.11887 (PMC8327967; doi:10.7717/peerj.11887)
Supplement: Supplemental Information 5 [file peerj-09-11887-s005.docx]

| The parameter settings of HMMCAS | | |
| --- | --- | --- |
| Description | Sequence | Hit |
| Significance E-value threshold | 0.01 | 0.03 |
| Report E-value threshold | 1 | 1 |
